# Correction to: Probabilistic solvers enable a straight-forward exploration of numerical uncertainty in neuroscience models

**DOI:** 10.1007/s10827-023-00856-w

**Published:** 2023-06-26

**Authors:** Jonathan Oesterle, Nicholas Krämer, Philipp Hennig, Philipp Berens

**Affiliations:** 1 Institute of Ophthalmic Research, University of Tübingen, Tübingen, Germany; 2 Department of Computer Science, University of Tübingen, Tübingen, Germany; 3 Max Planck Institute for Intelligent Systems, Tübingen, Germany; 4 Tübingen AI Center, University of Tübingen, Tübingen, Germany

**Correction to: Journal of Computational Neuroscience**

10.1007/s10827-022-00827-7

In the published article, the authors noticed that there is an error in one of the equations.

In the paper Eq. 8 says:

$${t}_{spike}=\frac{{v}_{th}-v\left({t}_{i}\right)}{{t}_{i+1}-{t}_{i}}$$

However, it should simply be a linear interpolation, as indicated in the text:

$${t}_{spike}={t}_{i}+\left({t}_{i+1}-{t}_{i}\right) \frac{{v}_{th}-v\left({t}_{i}\right)}{v\left({t}_{i+1}\right)-v\left({t}_{i}\right)}$$

The original article has been corrected.

