# Supplementary material for: Probabilistic solvers enable a straight-forward exploration of numerical uncertainty in neuroscience models
Source: J Comput Neurosci. 2022 Aug 6;50(4):485–503. doi: 10.1007/s10827-022-00827-7 (PMC9666333; doi:10.1007/s10827-022-00827-7)
Supplement: Supplementary file 1 — Supplementary file1 (PDF 442 KB) [file 10827_2022_827_MOESM1_ESM.pdf]

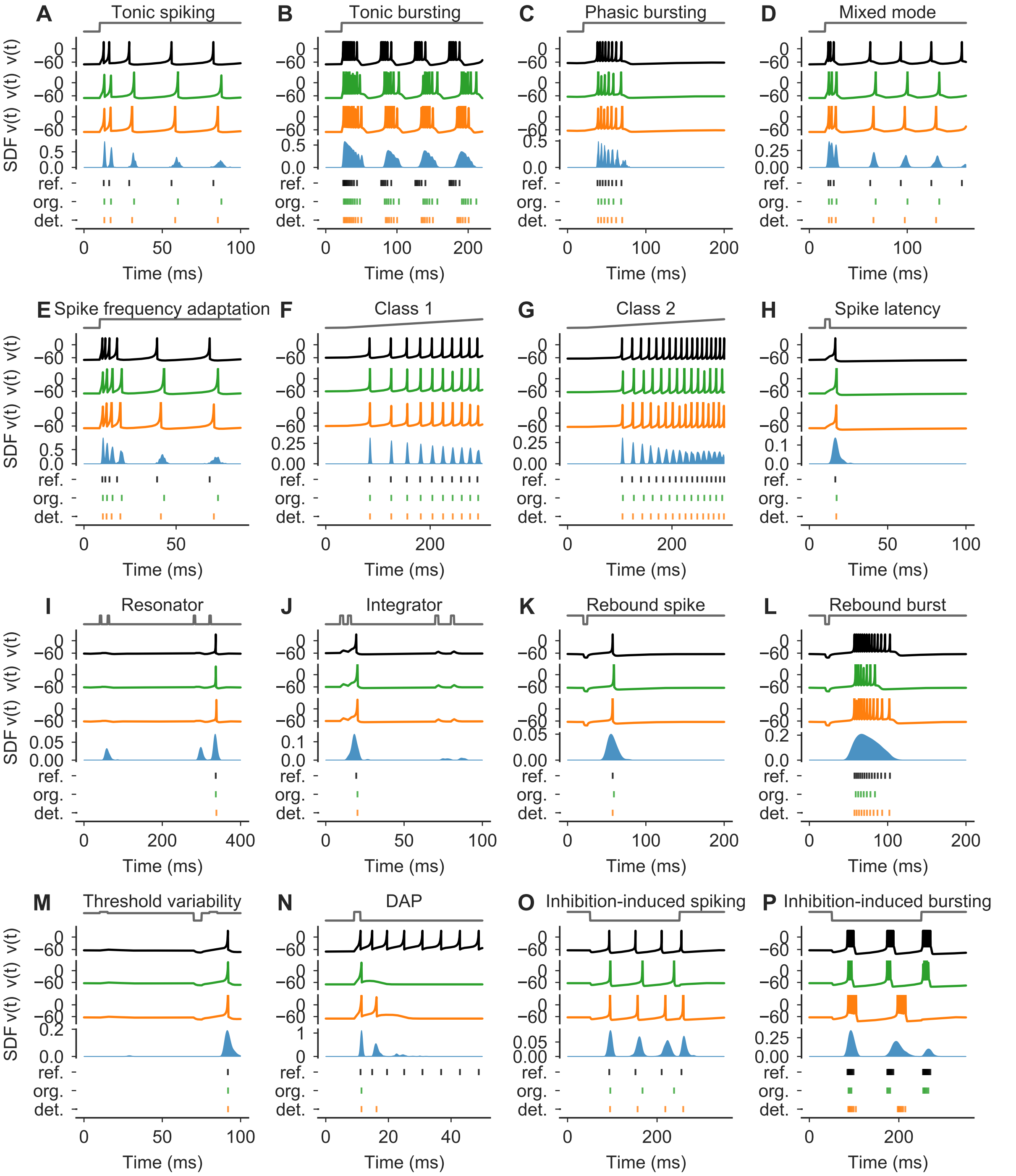

Fig. S1: **(A-P)** As in Figs. 2B and 2C: Simulations of the IN model for different parametrizations θ_i with stimuli I_{Stim} (normalized in gray). Solutions for $v(t)$ are shown for a reference solver (black), the original solver scheme (green) and a deterministic FE solver (orange). A SDF is shown for a FE$^{\ddagger}_{\ddagger}$ solver. For plotting, $v(t)$ were clipped at 30. In every panel, spike-times are shows in a raster plot for all deterministic solvers (bottom).
