# Supplementary material for: Probabilistic solvers enable a straight-forward exploration of numerical uncertainty in neuroscience models
Source: J Comput Neurosci. 2022 Aug 6;50(4):485–503. doi: 10.1007/s10827-022-00827-7 (PMC9666333; doi:10.1007/s10827-022-00827-7)
Supplement: Supplementary file 2 — Supplementary file2 (PDF 188 KB) [file 10827_2022_827_MOESM2_ESM.pdf]

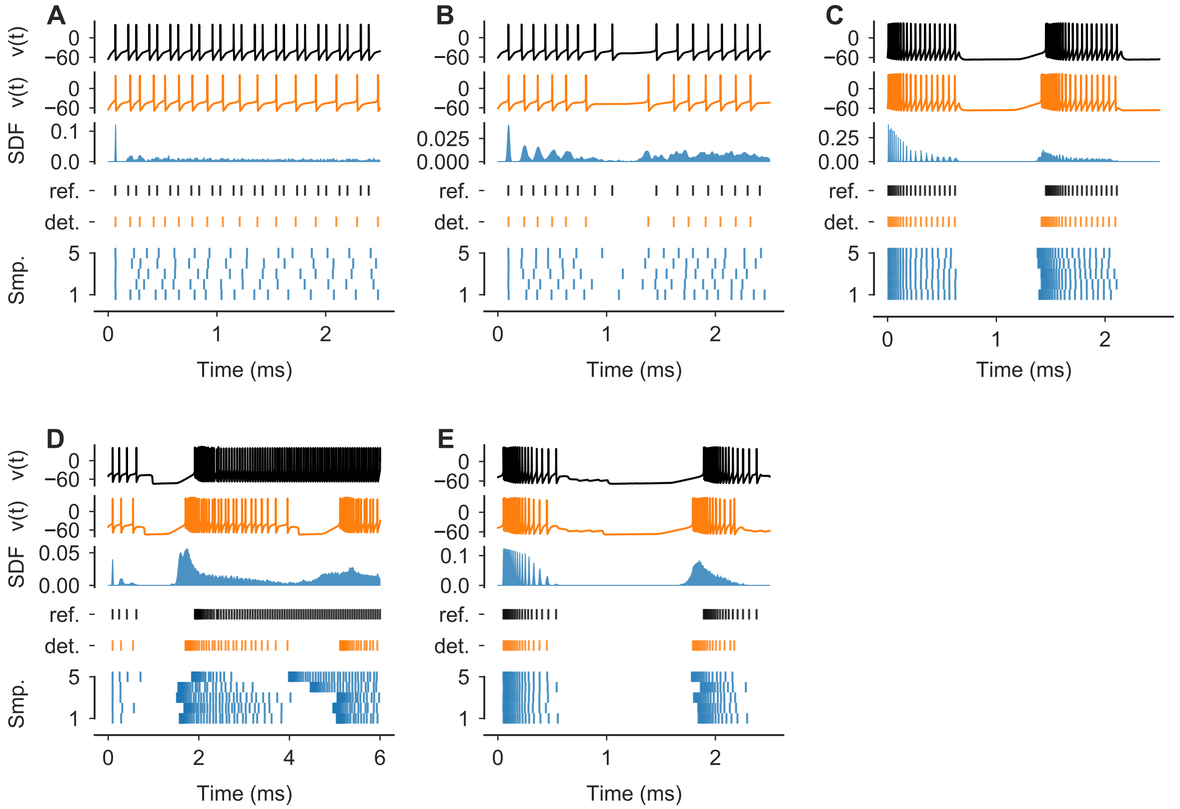

Fig. S2: **(A-E)** As in Figs. 2D and 2E: Simulations of the STG model for all five synaptic parametrizations (see Section C) from $\boldsymbol{\theta}^{\mathrm{a}}_{\mathrm{syn}}$ to $\boldsymbol{\theta}^{\mathrm{e}}_{\mathrm{syn}}$, respectively. Solutions for the membrane potential $v(t)$ of the LP neuron are shown for a reference solver (black) and a deterministic EE solver with $\Delta t = 0.1\,\mathrm{ms}$ (orange). A SDF is shown for a $\mathrm{EE}^{\ddagger}_{\mathrm{f}}$ solver using the same step-size. *Bottom panels*: Spike-times of the LP neuron are shown for the reference (black), the deterministic EE solution (orange) and for 5 samples from the probabilistic solver (blue).
