# Supplementary material for: Probabilistic solvers enable a straight-forward exploration of numerical uncertainty in neuroscience models
Source: J Comput Neurosci. 2022 Aug 6;50(4):485–503. doi: 10.1007/s10827-022-00827-7 (PMC9666333; doi:10.1007/s10827-022-00827-7)
Supplement: Supplementary file 3 — Supplementary file3 (PDF 43 KB) [file 10827_2022_827_MOESM3_ESM.pdf]

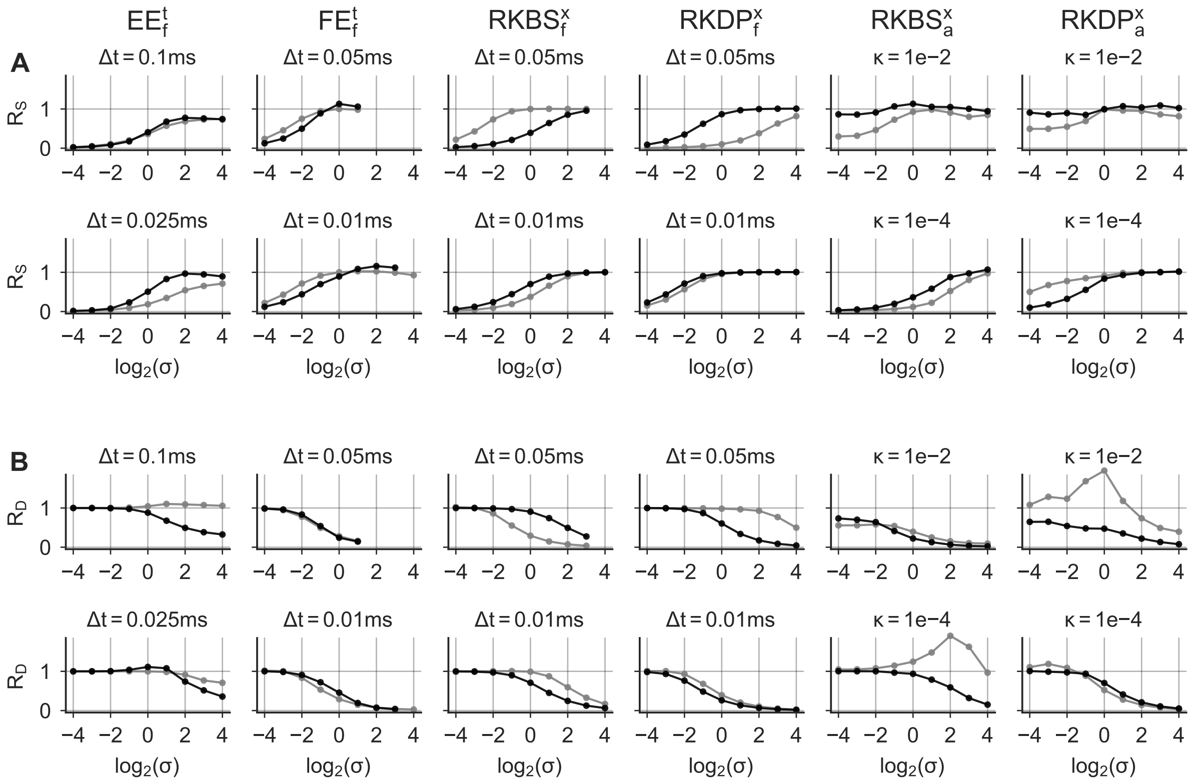

Fig. S3: **(A, B)** MAE ratios R_S and R_D for different solvers (column titles) and step-sizes Δt / tolerances κ (panel titles) for the step (grey) and the noisy step (black) stimulus as a function of the perturbation parameter σ (x-axis), respectively. See also Fig. 6D.
