# Supplementary material for: Probabilistic solvers enable a straight-forward exploration of numerical uncertainty in neuroscience models
Source: J Comput Neurosci. 2022 Aug 6;50(4):485–503. doi: 10.1007/s10827-022-00827-7 (PMC9666333; doi:10.1007/s10827-022-00827-7)
Supplement: Supplementary file 4 — Supplementary file4 (PDF 402 KB) [file 10827_2022_827_MOESM4_ESM.pdf]

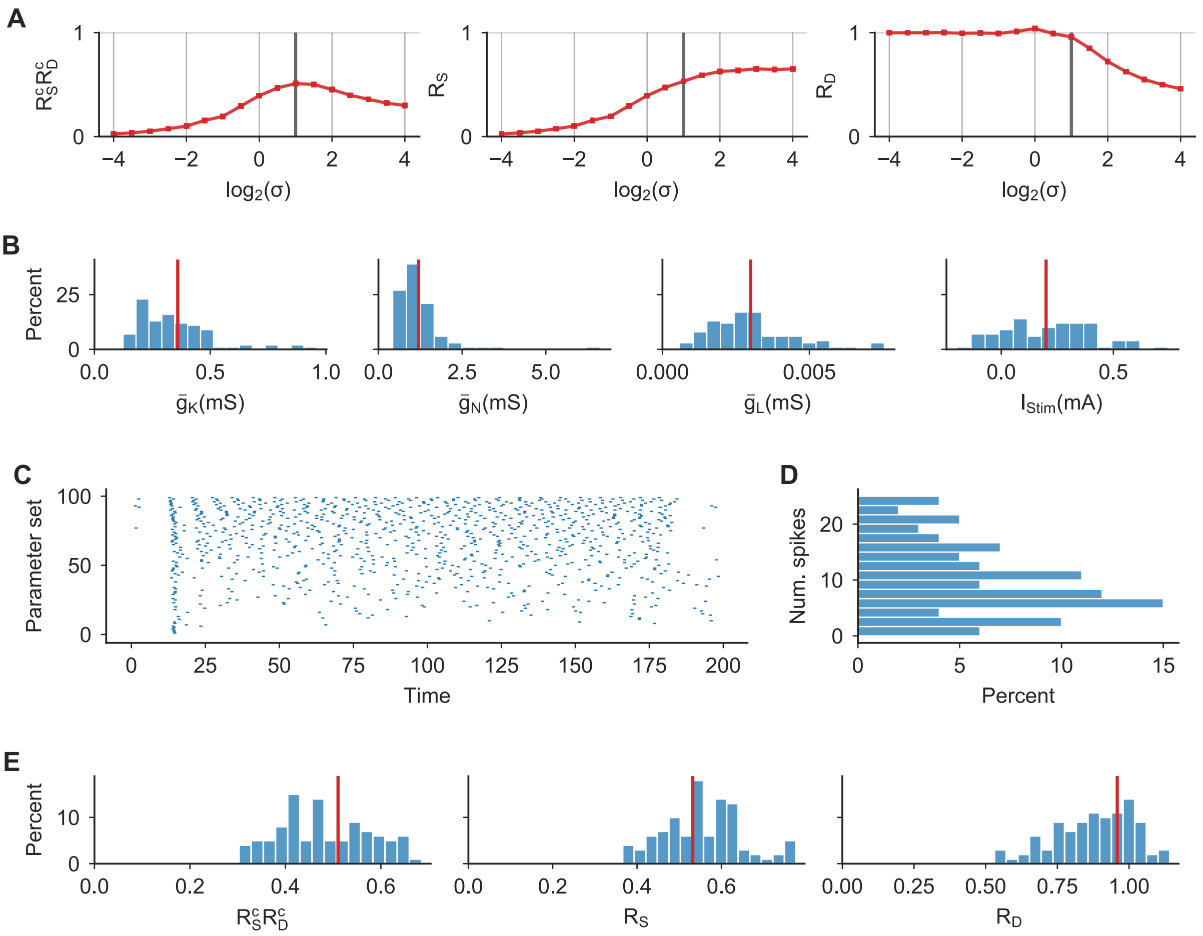

Fig. S4: Simulations of the classical HH model for the noisy step stimulus computed with a $\mathrm{EE}^{\mathrm{t}}_{\mathrm{f}}$ solver using $\Delta t = 0.1$ ms, a deterministic version of this solver and a reference solver. Solutions were computed for different HH model parameter sets ($\bar{g}_{\mathrm{K}}$, $\bar{g}_{\mathrm{N}}$, $\bar{g}_{\mathrm{L}}$, I_{Stim}). The values of the noisy step stimulus were independently sampled for every parametrization. The stimuli were constructed as described in Section 2.5.2, but with only 20 knots and different amplitudes to create a larger variety of spiking patterns. For every parametrization, the stimulus amplitudes at the knots were drawn from a uniform distribution between $I_{\mathrm{Stim}} - 0.2\,\mathrm{mA}$ and $I_{\mathrm{Stim}} + 0.2\,\mathrm{mA}$. **(A)** Calibration for the default HH model parameters (see Section C) as a function of the perturbation parameter σ measured as $R^c_S R^c_D$, R_S and R_D. For each σ, 100 samples were computed. The perturbation parameter resulting in the best calibration $\hat{\sigma}$ (largest $R^c_S R^c_D$) is highlighted (gray vertical line). **(B)** 100 HH model parameters sets drawn from log-normal distributions for the conductances and from a normal distribution for I_{Stim}. The means of all distributions were equal to the default parameters (red vertical lines). **(C, D)** Raster plot of spike-times and the distribution of the number of spikes for all 100 model parameter sets computed with the reference solver, respectively. The samples in the raster plot were sorted by the number of spikes. **(E)** $R^c_S R^c_D$, R_S and R_D for all 100 model parameter sets computed by generating 100 samples (using the optimized perturbation parameter $\hat{\sigma}$ from (A)), a deterministic solution and a reference solution for each set. The respective values for the default HH model parameters are shown for comparison (red vertical lines).
